# Mesoporous Carbon Composites Containing Carbon Nanostructures: Recent Advances in Synthesis and Applications in Electrochemistry

**DOI:** 10.3390/ma17246195

**Published:** 2024-12-18

**Authors:** Agnieszka Hryniewicka, Gabriela Siemiaszko, Marta E. Plonska-Brzezinska

**Affiliations:** Department of Organic Chemistry, Medical University of Bialystok, Mickiewicza 2a, 15-222 Bialystok, Poland; gsiemiaszko@gmail.com

**Keywords:** mesoporous carbon, composite, carbon nanostructure, electrochemistry, electrocatalysis

## Abstract

Carbon nanostructures (CNs) are various low-dimensional allotropes of carbon that have attracted much scientific attention due to their interesting physicochemical properties. It was quickly discovered that the properties of CNs can be significantly improved by modifying their surface or synthesizing composites containing CNs. Composites combine two or more materials to create a final material with enhanced properties compared with their initial components. In this review, we focused on one group of carbon materials—composites containing CNs (carbon/CN composites), characterized by high mesoporosity. Particular attention was paid to the type of synthesis used, divided into hard- and soft-templating methods, the type of polymer matrix precursors and their preparation method, heteroatom doping, pore formation methods, and correlations between the applied experimental conditions of synthesis and the structural properties of the composite materials obtained. In the last part, we present an updated summary of the applications of mesoporous composites in energy storage systems, supercapacitors, electrocatalysis, etc. The correlations among porous structures of materials, heteroatom doping, and electrochemical or catalytic efficiency, including activity, selectivity, and stability, were also emphasized. To our knowledge, a single review has never summarized pyrolyzed mesoporous composites of polymer-CNs, their properties and applications in electrochemistry.

## 1. Introduction

Carbon nanostructures (CNs) are a diverse family low-dimensional allotropes of carbon, including carbon black (CB), carbon nanofibers (CNF), fullerene, graphene, and larger structures, such as carbon nanotubes (CNTs), carbon nano-onions (CNOs), and carbon nanohorns (CNHs). These CNs possess unique chemical, physical, and mechanical properties, which have attracted the attention of scientists. Among these, graphene’s two-dimensionality stands out, resulting in exceptional high tensile strength, transparency, and electrical conductivity. These properties have led to the application of carbon nanomaterials in a wide range of fields, from sensing transducers to conducting materials, electronics, catalysis, and advanced biochemical sensors, showcasing their impressive utility [1].

It was quickly discovered that the properties of CNs could be improved by surface modification or the synthesis of composites containing CNs. The surface functionalization of CNs has the advantages of a large surface area, solid binding capability, improved dispersibility, and high mechanical strength. These features ensure the excellent performance of CNs but also ensure great potential as building blocks for hybrid carbon-based nanomaterials [2]. The resulting composites, with their significantly improved properties, offer a promising outlook for the future of materials science and nanotechnology [3]. For example, constructing porous carbon structures by introducing sp^2^ carbon structures, i.e., carbon fiber or graphene, has demonstrated superior performance in terms of conductivity enhancement [4,5].

Furthermore, graphene quantum dots combined with hydrogels have new possibilities for their applications in medicine as highly effective and long-lasting anticancer agents [6]. Composite materials have applications mainly in electronics, sensors, and tissue engineering. The recent development of quenched carbon and activated graphene proves that discoveries with a wide range of applications are still possible in this field [3]. Notably, the carbon/CN composites obtained through the annealing of polymer–carbon hybrids constitute an essential group of CN-based composites.

Among the composites, mesoporous materials constitute a significant group of materials. Mesoporous carbon materials with highly hierarchical arrangements have gathered great attention due to their uniform porosity, high surface area, shape selectivity, high-volume storage capability, enhanced mass transport, and diffusion. Various fields of application of these materials include separation, adsorption, photocatalysis, photovoltaic solar cells, energy conversion and storage, drug delivery, and chemical sensing [7]. The International Union of Pure and Applied Chemistry (IUPAC) classification of porosity describes mesopores as pores of sizes ranging from 2 to 50 nm. This range’s narrow distribution of pore sizes provides unique properties and many application possibilities [8].

This review describes mesoporous carbon materials based on composites containing CNs (carbon/carbon nanostructure composites; carbon/CN composites). We were interested only in the advantages of enriching CNs’ ‘pure’ porous carbon and their applications in various fields. Hence, examples covering metal-based carbons were not included. The large number of publications on graphene and CNTs has led us to limit the reports to the ones reported in 2016–2024. For other nanostructures, all related publications were cited. When describing the materials, special attention was paid to the type of polymeric matrix precursor and the method of its preparation, such as the pore-forming method, the temperature of pyrolysis, the surface area, the pore volume and size, heteroatom doping, application, and specific features. The subsections are divided according to the type of application. To our knowledge, a single review has never summarized pyrolyzed polymer-CN composites. This review supplements the existing reviews on mesoporous carbon materials [7,8,9,10,11,12] and composite materials containing CNs [3,13,14,15,16,17].

## 2. Definition of Mesoporous Materials and Composites

Mesoporous carbon is a solid material with a unique combination of ordered and disordered networks and a pore size distribution ranging from narrow to broad, which stands out from the IUPAC classifications. Unlike microporous (pore size < 2 nm) and macroporous materials (pore size > 50 nm), mesoporous carbon features a pore diameter of 2–50 nm [18,19]. A significant milestone in developing mesoporous materials was the synthesis of a new family of mesoporous silica (M41S) in 1992 by scientists at Mobil Corporation [20,21]. This breakthrough paved the way for the creation of the first highly porous mesoporous carbon, which was obtained by Ryoo et al. using a mesoporous silica template, and this achievement gave rise to the development of this field of science and an increase in the number of publications [22]. Hierarchically organized mesoporous carbon materials have attracted the special attention of the modern scientific world due to their potential applications in many fields, including electrical double-layer capacitors (EDLCs), catalysis, wastewater treatment, and air purification, as well as the separation and adsorption of biomolecules [23,24,25,26,27,28].

Composite materials combine two or more materials to form a third material with improved properties. These materials have advantages over their components. A composite material’s performance is intricately tied to its design and synthesis. When carefully considered, a composite material can outperform its essential components. For example, incorporating graphene sheets into a composite material improves the electrochemical properties of the new material [29].

## 3. Physical and Chemical Properties of Mesoporous Materials with Relevance in Electrochemical Processes

Micro- and mesopores in the material provide a large surface area and total pore volume, which, in turn, provides easy access by molecules to active sites in the entire volume of the material. Catalytic reactions occur in the whole volume of the material, not only on its surface, which effectively affects the efficiency of the catalytic reaction [9]. Diffusing molecules or electrolyte ions can easily penetrate mesopores. Their size can be easily tuned to the size of the tested molecule, thus influencing the increase in the efficiency of the catalytic reaction. Meanwhile, materials dominated by micropores have disadvantages, mainly including slow mass transport, an unstable carbon skeleton at high temperatures, and limited electrical conductivity. Micropores also constitute a physical barrier to transporting the tested molecules into the porous material. As a result, catalytic reactions using microporous materials take place mainly on their surface, limiting the efficiency of the catalytic reaction. Macroporous CNs usually have small surface areas and lack pore size selectivity, which negatively impacts the value of the achieved catalytic reaction efficiency [8].

Mesoporous CNs constitute a large family of porous materials with improved physicochemical properties compared with micro- and macroporous materials. These include improved electrical and thermal conductivity, surface hydrophobicity, and low density. In addition, the hierarchical organization of pores, the chemical and thermal stability, the inertness, and its biocompatibility make mesoporous carbon suitable for many applications [10,19].

## 4. Preparation of Mesoporous Carbon/CN Composites

### 4.1. Possible Modification Approaches of CNs

Carbon/CN composites have been prepared using pristine or modified CNs. Depending on the organic network’s synthesis, pristine CNs have been dispersed in a reaction mixture by simply sonication in the liquid medium [30], dispersed by applying an appropriate surfactant aqueous solution subjected to ultrasonication [31], or mixed by mechanical milling [32]. Later on, these dispersed CNs may be combined with substrates of the organic network [32] or undergo the following phenomena: (1) physical adsorption of the substrates on their surface [33], as well as (2) the formation of covalent bonds with substrates bearing an appropriate functional group (e.g., polymers terminated with azide forming aziridine rings with double bonds of CNOs) [34]. Functionalization of CNs includes (1) surface oxidation [35] and (2) covalent linkage to small organic molecules bearing functional groups involved in the synthesis of the organic networks [34]. An example of surface oxidation is the surface-induced assembly strategy reported by Tu et al., where negatively charged multiwalled carbon nanotubes (MWCNTs) caused the assembly of colloidal SiO_2_ nanoparticles (a pore-forming agent) on their surface [35]. An illustration of the covalent linkage between organic networks and CNs is found in research conducted by our group. Modified CNOs catch the dithiocarbonate moiety and serve to create polymer chains of a controlled length via so-called RAFT/MADIX (reversible addition–fragmentation chain-transfer/macromolecular design by interchange of xanthates) polymerization [34].

### 4.2. Methods of Synthesis of Mesoporus Carbon Composites

**Figure 1:**
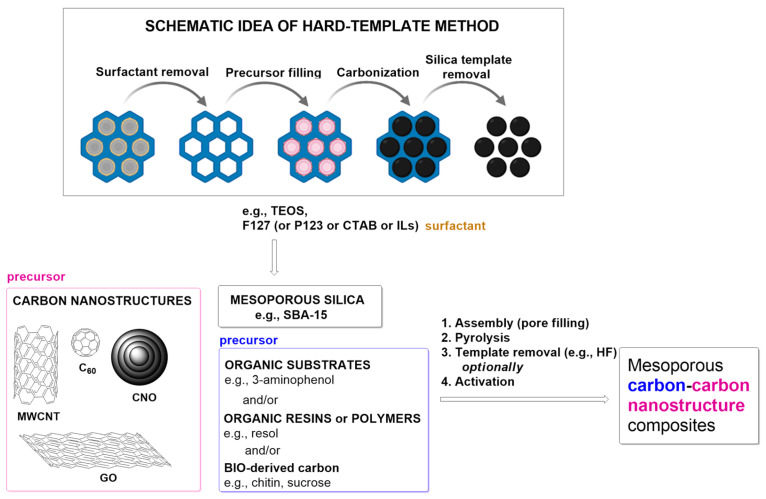
Schematic idea of hard-template approaches and their application to the synthesis of mesoporous carbon/CN composites. Abbreviations: TEOS—tetraethyl orthosilicate; F127 and P123—pluronics; CTAB—cetyltrimethylammonium bromide; ILs—ionic liquids; MWCNT—multiwalled carbon nanotube; GO—graphene oxide; CNO—carbon nano-onion.

The typical method of synthesizing mesoporous carbonaceous materials is a hard-template strategy (nanocasting) (Figure 1). The standard approach uses a mesoporous silica template (e.g., SBA-15) with no chemical interactions between the template and the carbon-sourcing components. A significant disadvantage of this method is the necessity of using aggressive acidic or alkaline conditions to remove inorganic templates, resulting in the material’s disorganized porosity and structure. The main steps of the procedure involve (1) preparation of the silica template, (2) filling of the template with carbon precursor and appropriate chemical reactions, (3) carbonization, and (4) template removal, affording porous carbon material [19,36]. Silica nanoparticles may be polymerized in situ on the surface of CNs (in the present case, CNTs) using tetraethyl orthosilicate (TEOS) [37]. Another inorganic hard template frequently used is the MgO particle [38].

In a synthesis of mesoporous carbon/CN composites (Figure 1), commercially available SBA-15 [39,40,41,42] or tetraethyl orthosilicate without [37,43] or with a proper surfactant such as pluronic F-127 [44,45,46], pluronic P123 [47], or cetyltrimethylammonium bromide (CTAB) [48] or an ionic liquid ([C_18_Mim]Br) [49] as the silica source are applied. Then the CN and carbon precursors, including organic substrates such as 3-aminophenol [43], pyrrole [47], dicyandiamide [45], aniline [35], urea [46], and ethylenediamine [37] etc. and bio-derived products like sucrose [42] or resins (e.g., resol [37,46]), are used to fill the pores in the assembly process (e.g., evaporation-induced self-assembly (EISA) [44,46]. After pyrolysis, the template should be removed with hydrofluoric acid [50].

**Figure 2:**
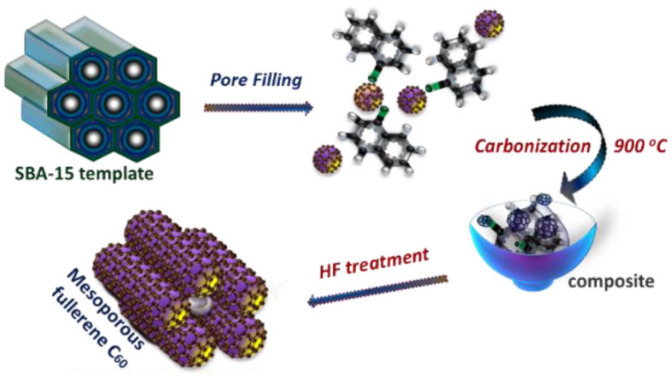
Example of the application of a hard-template approach. Synthesis of polymerized mesoporous C_60_ [40].

The materials obtained in this way can be activated (e.g., with KOH [51] or K_2_CO_3_ [52]). An example of applying the hard-template method for synthesizing mesoporous carbon/CN composites can be the preparation of the mesoporous fullerene C_60_ (Figure 2) [50]. In this process, C_60_ and 1-chloronaphthalene are used as precursors and mesoporous SBA-15 silica is used as a matrix. The first synthesis of mesoporous carbon/graphene composites dates back to 2010, when Yang et al. reported a bottom-up hard-template route to prepare 2D graphene-based nanosheets with a sandwich structure [53]. Then Lei et al. described a preparation of 3D graphene-based mesoporous carbon composites using a hard-template approach [50].

Table 1 summarizes the advantages and disadvantages of the hard- and soft-template methods. It should be noted that hard-template methods involve time-consuming silica removal procedures, limiting their further industrial use. Therefore, the soft-template methods, which have the advantages of easy processing, high efficiency, and low cost, are widely employed to synthesize nanocarbon–porous carbon composites. The main advantage of the soft-templating approach is the lack of harsh conditions to remove the inorganic templates and greater porosity control [9].

**Figure 3:**
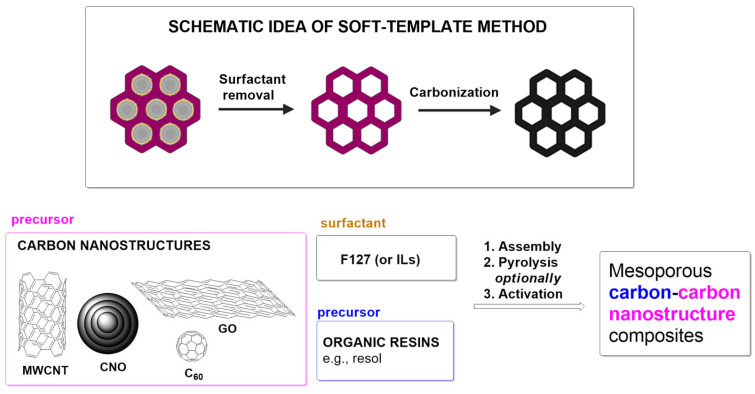
Schematic of soft-template approaches and their application to the synthesis of mesoporous carbon/CN composites. Abbreviations: MWCNT—multiwalled carbon nanotube; GO—graphene oxide; CNO—carbon nano-onion; F127—pluronic; ILs—ionic liquids.

**Figure 4:**
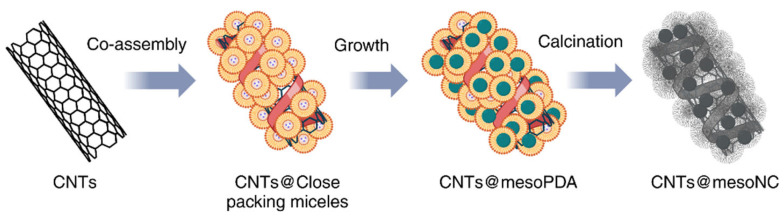
Example of the application of a soft-template approach: the synthesis of CNTs from mesoporous N-doped carbon core-shell nanofibers.

Typically, polymerization of cross-linked resin is carried out in the presence of a self-assembled amphiphilic block copolymer (surfactant). The process starts with (1) the self-assembly of micelles of the surfactant and resin precursors and thermopolymerization, followed by (2) removal of the soft template, and (3) carbonization [54]. In synthesizing mesoporous carbon/CN composites (Figure 3), pluronic F-127 [23,51,52,55,56,57] is often used as a surfactant. The carbon precursors are CNs and resins (e.g., phenol-formaldehyde [51,52,57] and resorcinol-formaldehyde [23]). An example of applying the soft-template method for synthesizing the mesoporous carbon/CN composite is its preparation from CNTs and mesoporous N-doped carbon core-shell nanofibers (Figure 4) [58]. The first report of using a soft-template route to synthesize mesoporous carbon/graphene composite was reported by Wang et al. [59].

Similar to soft-templating is the block copolymer templating approach. The ease of manipulating copolymers’ chemical and physical properties and their ability to assemble into domains make them a great precursor of porous materials. Copolymers are typically built of pore-forming sacrificial blocks, which create the pores in the material, and carbon-sourcing blocks, which provide the carbon structure [60]. For example, our group described the synthesis of 6-*star*-(polymethyl acrylate-*b*-polyacrylonitrile) polymers and their application to prepare CNO-polymer composites. During mesoporous carbon manufacturing, the composite undergoes thermal treatment, in which self-assembled polyacrylonitrile domains form graphene nanosheets, and poly(methyl acrylate) domains are thermally depolymerized [34].

### 4.3. Factors Affecting the Size of the Pores

The size of the pore is strictly affected by the synthesis conditions. Key factors include the design of carbon precursors, the structure-directing agent, and the solvent. Furthermore, it is essential to choose the proper stoichiometry and carbonization temperature [61]. In the case of hard-template methods, the pores’ size in the obtained material is determined by the wall thickness of the inorganic matrices, which depends on the synthesis conditions [62]. In particular, manipulating the micelles’ size in materials synthesized by soft-template methods can affect pore diameters. Mai and co-workers prepared mesoporous N-doped carbon nanospheres with controlled pore diameters using block copolymer PEO_114_-*b*-PS_n_ (polyethylene oxide and polystyrene) by manipulating the polystyrene chains’ length. The obtained pore sizes depended on the length of the PS block; thus, using 60 styrene units, the pore diameter in the resulting carbon material was 8 nm, and when PS with a length of 390 units was used, the pore diameter increased to 33 nm. The authors found a linear dependence of d_meso_ on the square root of n (R^2^ = 0.98) [63]. In addition, the pore size can be adjusted by mixing swelling agents (pore expanders) and micelle-forming polymers (e.g., pluronics). The swelling agent and the polarity of the polymer (in particular, the lipophilic moiety) must be matched for hydrophobicity. For example, by using 1,3,5-trimethylbenzene, the diameter of mesopores could be increased from 5.9 to 8.1 nm. Using dioctyl phthalate enabled access to mesopores up to 13.5 nm [61]. It should be noted that the presence of swelling agents can affect the pore size and the mechanism of nanostructure formation [64].

By changing the reaction’s stoichiometry, e.g., the ratio of the carbon precursor to the structure-directing agent, the pore size in the material can be controlled. Larger pore diameters are noticeable as the carbon precursor content decreases relative to the structure-directing agent. For example, when the carbon precursor to structure-directing agent ratio was 0.83, the pore diameter was about 7 nm, while when the ratio was 0.25, the diameter increased to 44 nm. However, it should be noted that although the pore size can be manipulated this way, it is often accompanied by a change in the type of pore arrangement (i.e., 2D hexagonal and cubic symmetry) [65].

An analysis of the effect of the carbonization temperature on pore size reveals a significant trend: the number of micropores increases with higher carbonization temperatures. Zhao et al. obtained resole/pluronic F127-based carbon materials using different carbonization temperatures. The pore size values ranged from 6.8 nm (350 °C) to 2.2 nm (1400 °C). At a higher temperature, the pore size distribution was slightly wider, and the surface gradually shrunk, the latter indicating partial pore collapse or micropore removal. However, the mesostructure remained remarkably well-preserved, providing reassurance about the stability of the carbon materials. Typically, higher temperatures lead to mesopores with a smaller diameter in the final carbon material. The destruction of the ordered structure at high carbonization temperatures can lead to pore collapse or excessive micropore formation [66].

### 4.4. Heteroatom-Doped Mesoporous Carbon/CN Composites

**Figure 5:**
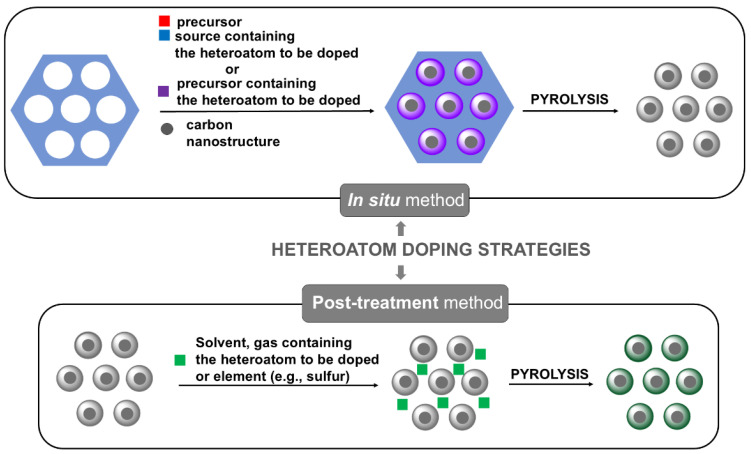
Heteroatom doping strategies for the synthesis of nanostructure carbon/CN composites.

Typically, doping with N, S, and P is carried out to improve mesoporous CNs’ electrochemical and catalytic performance. Heteroatom doping is usually realized by two different strategies using in situ or post-treatment methods (Figure 5). The in situ doping method is also called the direct synthesis approach. Heteroatoms are introduced by utilizing N- or S-containing carbon precursors. The S atoms may be acquired from synthetic (2-thenaldehyde) [67] or natural (macadamia nutshells) [68] sources.

The in situ method relies on the thermal treatment of the precursor containing heteroatoms or the precursor with the source of heteroatoms with templates to form a well-ordered intermediate composite, followed by pyrolysis. This doping method is beneficial for embedding the entire carbon matrix with homogeneous foreign atoms [69]. Another method used to obtain heteroatom-doped materials is a post-treatment process through oxidation, thermal polymerization, and replacement reactions. The post-treatment methods can be further divided into dry and wet modifications. For the dry modification, the carbon material is treated with a heteroatom-containing gas (e.g., ammonia to obtain N-doped materials) at high temperatures without solvents [70]. The post-treatment method with the elemental S of graphene-based carbon nanosheets mixes the material with S, with subsequent heating at 155 °C [71]. For the wet modification, the carbon material is usually impregnated in the heteroatom-containing chemical solutions for several minutes, followed by drying and heating treatment [69].

## 5. Application of Mesoporous Carbon/CN Composites: Catalysis and Electrochemistry

### 5.1. Electrochemical Applications of Mesoporous Carbon/CN Composites

**Figure 6:**
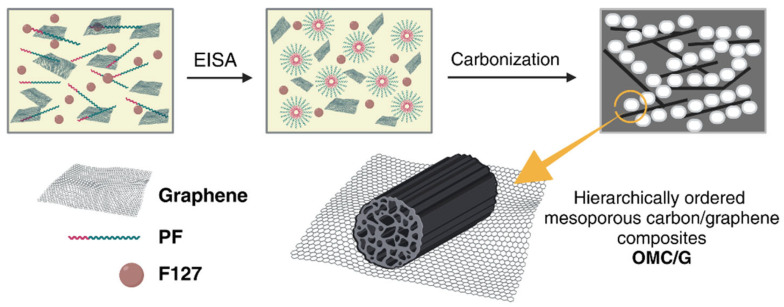
Preparation of ordered mesoporous carbon/graphene composites.

CNs, like graphene, can be used as a supercapacitor electrode thanks to their large surface area, excellent electron mobility, and large potential window [72]. However, individual graphene nanosheets are aggregated due to strong van der Waals forces that interfere with the rapid diffusion of ions. An effective way to prevent sheet aggregation and, consequently, to improve the energy storage efficiency of graphene-based materials is to introduce mesoporous carbon into graphene sheets [73]. Carbon/CN composites have a structure with high mesoporosity and electrolyte availability, making them an excellent material for electrochemical applications. Table 2 summarizes the mesoporous carbon-based composites that are used as supercapacitors. Most examples are related to graphene-based materials, especially those with the use of resols as a cheap, readily available source of mesoporous carbon. Examples include hierarchically ordered mesoporous carbon/graphene composites (OMC/G, Figure 6) made by solvent evaporation-induced self-assembly (EISA) [51].

Compared with OMC, the composite has a larger specific surface area (2109 m^2^ g^−1^). It achieves an exceptional capacitance of up to 329.5 F g^−1^ in an electrolyte of 6 M KOH at a current density of 0.5 A g^−1^. The inclusion of graphene in OMC/G composites significantly enhances the transport of electrons during charging and discharging processes due to its high conductivity, thus leading to excellent energy storage efficiency [51]. Chandrasekaran et al. demonstrated a novel and facile approach to producing resol-based graphene composites. This method relies on the gelation of an resorcinol-formaldehyde network by deprotonating the oxygen functionalities present in GO and subsequent carbonization. The resulting mesoporous carbon/graphene aerogels exhibit a high specific surface area, increased electrical conductivity, and higher specific capacitance than pristine graphene aerogels [74].

Another exciting report concerns the synthesis and thermal pyrolysis of a sulfonated graphene/N-doped mesoporous carbons composite (SG/NMC). This material possesses a specific surface area high up to 1040 m^2^ g^−1^ and exhibits a high specific capacitance of about 304 F g^−1^ at 1.0 A g^−1^, which is much higher than that of ordered mesoporous carbons (129 F g^−1^) and N-doped mesoporous carbons (223 F g^−1^). Moreover, the composite displays a capacitance of 209 F g^−1^ at a current density of 50 A g^−1^, and the capacity can be retained above 93% after 5000 cycles at 10 A g^−1^ [56]. Baruah et al. reported the synthesis of the mesoporous ternary nanocomposite by in situ polymerization of pyrrole (Py) in the presence of protonated g-C_3_N_4_ and rGO (rGO-pg-CN/PPyNTs). Although this material exhibits a low value of a specific surface area of 158 m^2^ g^−1^, its specific capacitance is high at around 800 F g^−1^ at a 0.5 A g^−1^ current density with 61% capacitive retention at sevenfold current density in a 1 M KCl electrolyte [75].

**Figure 7:**
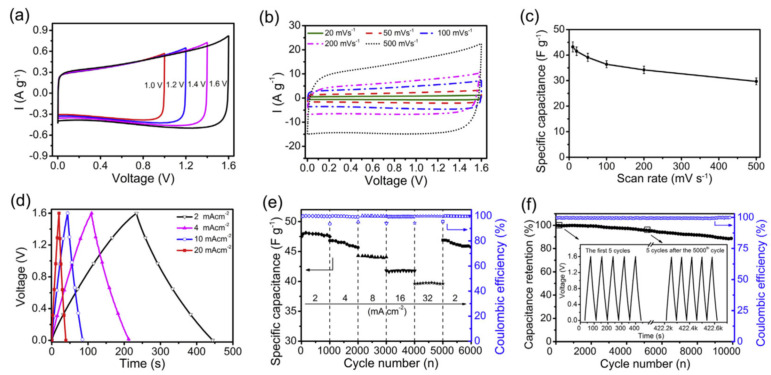
Electrochemical properties of the asymmetric capacitors in a PVA/LiCl gel electrolyte. (**a**) CV curves (10 mV s^−1^) at different potential windows. (**b**) CV curves at various scan rates. (**c**) Specific capacitance calculated from the CV curves as a function of the scan rate. (**d**) GCD curves at different current densities. (**e**) Rate performance at different current densities from 2 to 32 mA cm^−2^. (**f**) Long-term cycling stability at 10 mA cm^−2^. The inset is the GCD curves of the first 5 cycles and those after 5000 cycles [47].

Figure 7 shows the electrochemical properties of supercapacitors whose electrode material was graphene/N-doped ordered mesoporous carbon nanosheets (Table 2) [47]. The composite material was synthesized in situ by polymerization of Py directly on GO, giving PPy/rGO, which was further annealed and carbonized at 900 °C under an N_2_ atmosphere. The device delivered a specific capacitance of 196.5 F g^−1^ at 0.2 A g^−1^ in a 1 mol L^−1^ H_2_SO_4_ electrolyte. This device exhibited good capacitive behavior and fast charge–discharge performance as an asymmetric capacitor in a PVA/LiCl gel electrolyte, namely, a high energy density of 15.8 Wh kg^−1^ (1.01 mWh cm^−3^) and excellent long-term cycling stability (95.8% capacitance retention after 5000 cycles and 88.7% retention after 10,000 cycles).

**Table 2:** Applications of mesoporous carbon/CN composites as electrodes for supercapacitors.

Carbon Nanostructure	Precursor of Mesoporous Carbon	Template	Pore-Forming Agent	T (°C)	Surface Area (m^2^ g^−1^)	Pore Volume (cm^3^ g^−1^)	Pore Size (nm)	Heteroatom (%)	C*s* (F g^−1^)	Ref.
Graphene	Resol, urea	Hard	F127, TEOS	850	1348	1.18	3.6	N (3)	246	[46]
Graphene	3-Aminophenol, HCHO, ethylenediamine	Hard	TEOS	700	989	1.82	10.6	N (7)	249	[43]
Graphene	Pyrrole, (NH_4_)_2_S_2_O_8_	Hard	P123, TEOS	900	383	-	2–5	N (3.9)	196.5	[47]
Graphene	CCl_4_, ethylenediamine	Hard	SBA-15	600	362	0.43	4–22	N (10)	240	[39]
Graphene	Phenol, HCHO, S	Hard	F127, TEOS	800	1709	1.89	1.41 4.54	S (0.1)	314	[44]
Graphene	Resol, dicyandiamide	Hard	F127, TEOS	700	1569	1.38	0.56–6.4	N (6.25)	377	[45]
Fullerene C_60_	Chlorinated naphthalene	Hard	SBA-15	900	680	0.85	4.5–10.6	-	116	[40]
Graphene CNTs	Aniline, (NH_4_)_2_S_2_O_8_	Hard	SiO_2_	900	785.7	1.66	7–22	N (7.3)	112	[35]
Fullerene C_70_	Chlorinated naphthalene	Hard	SBA-15	900	585.8	0.79	2.7–10.1	-	172	[41]
Fullerene C_60_	Sucrose	Hard	SBA-15	900	808	1.5	3.6	-	213	[42]
Graphene	2,6-Diaminopyridine	Soft	PS-b-PEO	700	324	-	8–25	N (19)	256	[76]
Graphene	resorcinol, hexamine	Soft	F127	800	1072	-	2.7	-	209	[55]
Graphene	phenol, HCHO	Soft	F127	800	2109	1.24	3.41	-	329.5	[51]
Graphene	Resorcinol, mesitylene, hexamine	Soft	F127	800	1040	0.73	1.4–3.6	N (5.24)	304	[56]
Graphene	Phenol, HCHO	Soft	F127	800	1309	0.89	0.71–4.75	-	332.5	[52]
CNOs	‘S*tar*’ polymer, HCHO	Block copolymer	PMA chains	800	247	0.139	4–8	N (0.7)	139	[30]
CNOs	‘S*tar*’ polymer	Block copolymer	PMA chains	800	74	1.44	3–14	N (8.0)	83	[34]
Graphene	Pyrrole, (NH_4_)_2_S_2_O_8_	-	-	700	-	-	-	N (9)	296	[77]
Graphene	Glucose, graphene	-	-	800	763	3.06		-	305.5	[78]
Graphene	Ethylenediamine, phytic acid	-	-	900	596	0.55	3.7	N (3.6) P (0.3)	201	[79]
Graphene	Resorcinol, HCHO	-	-	800	534	1.28	7.2	-	120	[74]
Graphene	CTAB, aqueous mesophase pitch	-	-	900	1151	0.86	4.3–17	-	356.3	[80]
Graphene	Polyacrylonitrile	-	-	800	389	-	15–65	N (10)	431.9	[81]
Graphene	Urea, pyrrole	-	-	550	158	-	3.2	N (-)	803	[75]
Graphene CNTs	Activated carbon	-	-	450	953	1.075	4.5	N (7.38)	750	[82]
CNOs	Resorcinol, HCHO	Soft	F127	800	723	0.659	5.5–6.5	-	85	[83]
CNOs	Resorcinol, HCHO, melamine	Soft	F127	800	923	1.242	8–11	N (2.3)	160	[83]

Abbreviations: Cs—specific capacitance; TEOS—tetraethyl orthosilicate; CTAB—cetyltrimethylammonium bromide.

Composites can be based on two different CNs, graphene and CNTs, as reported by Tu et al. Positively charged polyaniline served as the carbon source, and silica (SiO_2_) nanoparticles as the pore template, giving nanomaterials with precisely tunable dimensions (1D to 2D), diameters (35–210 nm), thicknesses (7–145 nm), and pore sizes (7–22 nm) [35]. Recently, Krestinin et al. described the carbon nanomaterial as mechanically strong, low-priced, and convenient for supercapacitors. It was obtained from resorcinol–formaldehyde xerogel and CNTs after carbonization at 800 °C. The specific surface area of the nanopaper exceeded 600 m^2^ g^−1^; the maximum capacitance was 155 F g^−1^ [84] in 1 M H_2_SO_4_ and 70 F g^−1^ in an organic electrolyte, such as a 1,1-dimethylpyrrolidinium tetrafluoroborate/acetonitrile solution [85].

Vinu et al., for the first time, reported the preparation of highly ordered mesoporous fullerene C_70_ materials using SBA-15 silica as a hard template [41]. The composite obtained in this way was characterized by a tunable porous structure and a controlled rod-shaped morphology, which significantly influenced the electrochemical properties of the obtained materials. Incorporating polymerized fullerene C_70_ in the structures enhanced the charge delocalization of carbon by introducing a more significant amount of the active sites in the electrocatalytic reaction. Additionally, the regulated porous structure of the material facilitated the access of the analyte to the active sites participating in oxygen reduction reaction (ORR). Consequently, the mesoporous C_70_ composite exhibited higher ORR activity than mesoporous carbon electrodes with a high specific surface area and limiting its current density, comparable with commercial 20% Pt/C. The same authors reported the preparation of ordered mesoporous fullerene C_60_/carbon hybrids by mixing the fullerene precursor in chloronaphthalene with different amounts of sucrose using SBA-15 as a template [42]. The addition of sucrose to the composite significantly increased the specific surface area of the obtained material, consequently influencing its electrochemical properties. The specific capacitance of the composite almost doubled (213 F g^−1^ at 0.5 A g^−1^) in comparison with the material obtained without the addition of sucrose (116 F g^−1^ at 0.5 A g^−1^). Improvement in the electrochemical properties for hybrid materials was also observed regarding Li-ion accommodation. The carbon layer on the C_60_ surface, formed by the pyrolysis of sucrose, prevented the irreversible reaction between Li^+^ ions and the electrolyte. The ordered mesoporous structure also ensured the easy transport of Li^+^ ions and their accommodation in active sites. The characterization data revealed that the prepared mesoporous hybrid materials might be used as supercapacitors and Li-ion battery electrodes.

**Figure 8:**
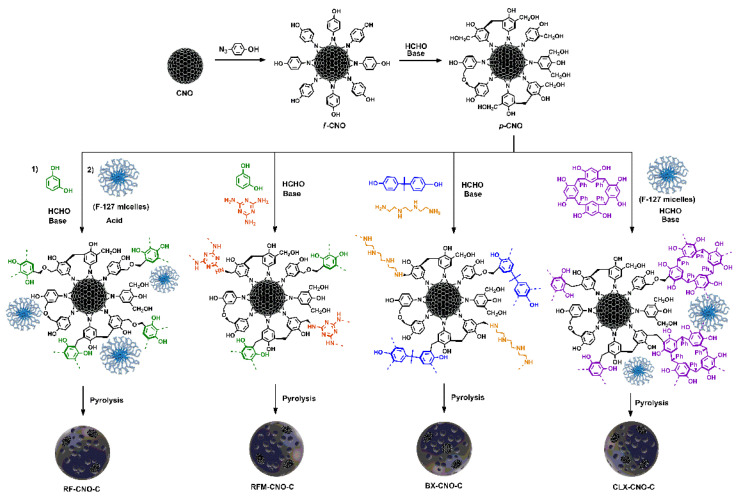
Composites containing CNOs and different resins [83].

Recently, we reported the synthesis of composites containing CNOs and resins (Figure 8), namely resorcinol–formaldehyde (RF-CNO-C), resorcinol–formaldehyde–melamine (RFM-CNO-C) benzoxazine made of bisphenol A and triethylenetetramine (BX-CNO-C), and calix[4]resorcinarene-derived (CLX-CNO-C) using pluronic F-127, and a subsequent carbonization process [83]. Adding CNOs to the materials significantly enhanced the textural properties of the composites, which were expressed by increasing the total pore volume (up to 0.932 cm^3^ g^−1^ for RF-CNO-C and 1.242 cm^3^ g^−1^ for RFM-CNO-C), which consequently significantly affected its electrochemical properties. The most optimal capacitive properties were shown by the carbon material derived from CNOs, resorcinol, and melamine, giving the highest specific capacitance of 160 F g^−1^ at a current density of 2 A g^−1^, which was stable after 3000 cycles.

The mesoporous carbon/CN composites were used as an attractive alternative to ion batteries due to their ordered mesoporous structure, rich edge defects, short diffusion pathway, and large interaction interface, allowing the insertion and extraction of the electrolyte ions [86]. These material properties proved particularly valuable in Li-S batteries (Table 3). Wang et al. proved that loading the materials with S can improve the conductivity of ions and reduce the ion transport resistance [87]. A mesoporous carbon/CN composite was synthesized using the carbonization process of ionic liquid 1-ethyl-3-methylimidazolium dicyanamide (Emim-dca) in the presence of MWCNTs. The obtained materials were used as electrodes in Li-S batteries. Outstanding battery performance parameters were achieved: the specific battery capacity reached 1558.6 mAh g^−1^ in the first cycle at 0.1 C. After 300 cycles at 1 C, the specific capacity decreased to 614.34 mAh g^−1^, and the attenuation was only 0.021% for each cycle. Post-thermal treatment has been widely applied to synthesize S-loaded carbon materials for Li-S batteries [88]. Wu et al. synthesized a composite material by combining N-doped carbon derived from chitin and g-C_3_N_4_, obtained by sequential dissolution methods and carbonization in the last step. The composite was used as a cathode material for a Li-S battery. When loaded with elemental sulfur, the fabricated cathode showed superior high-rate capability and remarkable cycling performance, which maintained a high reversible capacity of 1130 mAh g^−1^ after 500 consecutive electrochemical cycles [89]. Khan et al. revealed that N,S-co-doping of the materials results in lower charge transfer resistance and superior rate capability [71]. They fabricated N,S-co-doped carbon nanosheets anchored on the surface of rGO. The composite was prepared by polymerizing *m*-aminobenzene sulfonic acid on the GO’s surface and subsequent carbonization. The composite thus obtained contained an ordered porous structure (a homogeneous arrangement of micro- and mesopores in 3D), forming a free-standing electrode, facilitating the charging of S and the accommodation of unloaded polysulfides. A Li-S battery exhibited a stable specific capacity of 1355 mAh g^–1^ at 0.1 C and good rate performance. Furthermore, even at a high current density of 1 C (1 A g^–1^), it exhibited high storage with a capacity of 476 mAh g^–1^ over 300 cycles with a decay of 16.8% [71].

A chemical sensor can be defined as a device that continuously provides information about its environment, e.g., about the presence of a specific compound [100]. Analytes are often detected by converting their presence into electrical or optical signals. Mesoporous materials are ideal for constructing sensors due to their organized porous structure with active centers in the inner walls. This structure enables easy interaction with the analyte throughout the sensor’s volume, not just on its surface. Using mesoporous materials significantly optimizes the sensor’s parameters, particularly enhancing its sensitivity and increasing the detection limit of the induced signal. Electrochemical sensors (Table 4) are particularly attractive because of their remarkable detectability, experimental simplicity, and low cost. They occupy a leading position among the currently available sensors that have reached the commercial stage and have found a wide range of essential applications.

Zhu et al. synthesized hierarchical mesoporous B,N-co-doped carbon nanospheres with a core–shell structure immobilized on rGO [102]. The composite was applied as an electrochemical sensor of xanthine and guanosine. It was characterized by outstanding analytical parameters, including a considerably wide linear range of analyte determinations (xanthine (0.0915–103 μM) and guanosine (0.0822–128 μM)), excellent repeatability, good reproducibility, and stability.

**Figure 9:**
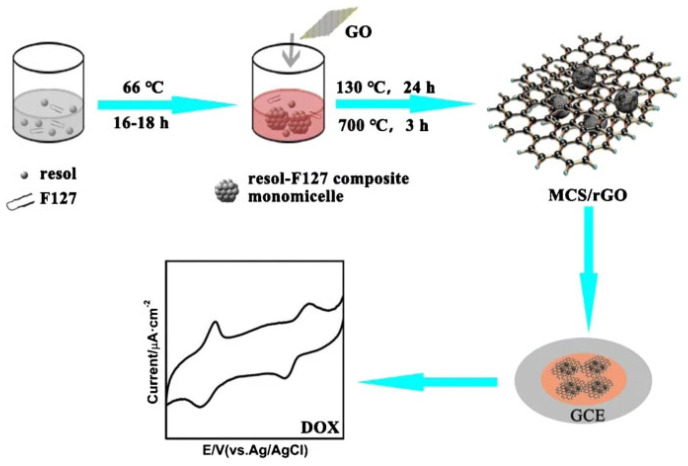
Preparation of an electrochemical sensor based on mesoporous carbon–graphene composites [57].

Another example of an effective electrochemical sensor was proposed by Liu et al. [57]. They described a modification of a glassy carbon electrode (GCE) with a composite of mesoporous carbon spheres and GO (Figure 9). It was applied to determine the cancer drug doxorubicin (DOX) in spiked serum. The π-interaction between DOX and rGO facilitated the adsorption of DOX. The modified GCE had a wide linear response range (10 nM–10 μM), a low limit of detection (1.5 nM), excellent selectivity, long-term storage stability, and reproducibility.

### 5.2. Catalytic and Electrocatalytic Properties of Mesoporous Carbon/CN Composites

Converting CO_2_ into valuable chemicals and low-carbon fuels is one effective way to reduce CO_2_ emissions and promote gas recycling. Electrochemical CO_2_ reduction (ERC) has significant economic potential due to its ability to convert renewable energy into gaseous and liquid fuels. Doping mesoporous carbon materials with CNTs or CNHs increases the electrical conductivity, improving electrochemical measurements. The presence of mesopores in the tested composites increases the electrocatalytic activity in both reactions, namely the electroreduction of oxygen and CO_2_ [104,105].

**Figure 10:**
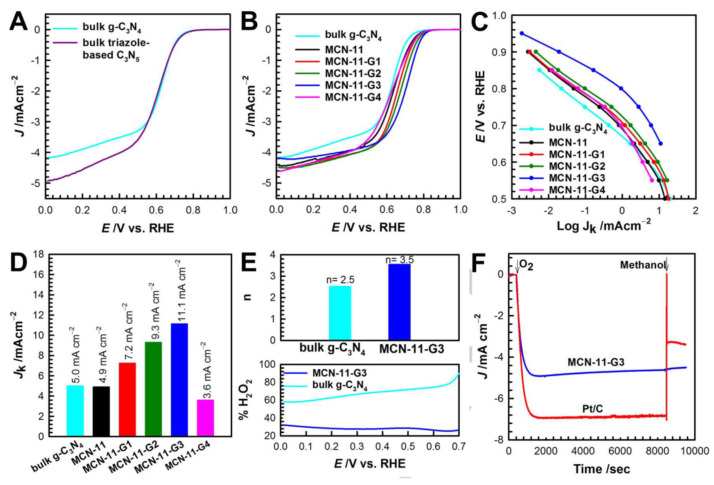
(**A**) The LSV of bulk g-C_3_N_4_ (cyan) and bulk triazole-based C_3_N_5_ (purple). (**B**) The LSV and (**C**) Tafel plots and (**D**) kinetic current density of MCN-11 (black), MCN-11-G1 (red), MCN-11-G2 (green), MCN-11-G3 (blue), MCN-11-G4 (pink), and bulk g-C_3_N_4_ (cyan) measured in an O_2_-saturated 0.1 M KOH electrolyte at 5 mV s^−1^ and 1600 rpm. (**E**) (top) Electron transfer number and (bottom) HO_2_^−^ yield of bulk g-C_3_N_4_ and MCN-11-G3 and (**F**) chronoamperometric responses at 0.56 V in N_2_-saturated 0.1 M KOH on MCN-11-G3 (blue) and Pt/C electrode (red) following the introduction of O_2_ and methanol (0.3 M) at a 1600 rpm rotation rate. Abbreviations: MCN-11; MCN-11-G1; MCN-11-G2; MCN-11-G3, and MCN-11-G4—the materials obtained for the g-C_3_N_4_ hybrids prepared from GO with different amounts: 0, 0.7, 1.4, 2.1, and 2.8 wt%, respectively [109].

N-doped graphene has been verified as an efficient ORR electrocatalyst via a four-electron transfer pathway [106]. The stacking of graphene sheets via strong van der Waals force reduces the surface area, porosity, and the number of reagent adsorption sites in N-doped graphene [107]. In addition, it results in impaired mass transport of the reactants or products, resulting in a loss of electromotive force and inhibiting the performance of energy-conversion cells. Adding N-doped graphene to other porous structures, forming a new composite, is one of the promising approaches to prevent deterioration of the activity and improve the ORR’s electrocatalytic activity [108]. Kim et al. reported the preparation of mesoporous carbon nitrides (obtained via simple self-assembly of 5-amino-1*H*-tetrazole) hybridized with graphene using graphene–mesoporous silica hybrids as a template [109] (Table 5). The ORR activity of bulk g-C_3_N_4_ and bulk triazole-based C_3_N_5_ were investigated using linear sweep voltammetry (LSV) (Figure 10). The studies showed that introducing triazole-based C_3_N_5_ moieties to the materials leads to a higher diffusion-limiting current density than the pristine g-C_3_N_4_. This property results mainly from the strong adsorption of O_2_ on active carbons (C1 and C4 neighboring the N-N bond) in the triazole moiety and increasing conjugation inside the g-C_3_N_4_ matrix due to the presence of the sp^2^-hybridized N of triazoles that leads to fluent 4 e^−^ transfer in the ORR. Additionally, the overpotential of the composites significantly decreased due to their 3D mesoporous structure and the strong electronic coupling between triazole-based mesoporous C_3_N_5_ and graphene, improving the ORR’s kinetics. The best electrocatalytic properties regarding the ORR are shown by MCN-11-G3 (the composite prepared from g-C_3_N_4_ and GO with 2.1 wt%), which presents a 3.5 electron transfer number with a peroxide generation yield of 23% (Figure 10C,D). Although the ORR activities of MCN-11-G3 are lower than those of Pt/C, this composite has an excellent tolerance of methanol (Figure 10F), allowing it to be used as an electrocatalyst for methanol fuel cells.

**Figure 11:**
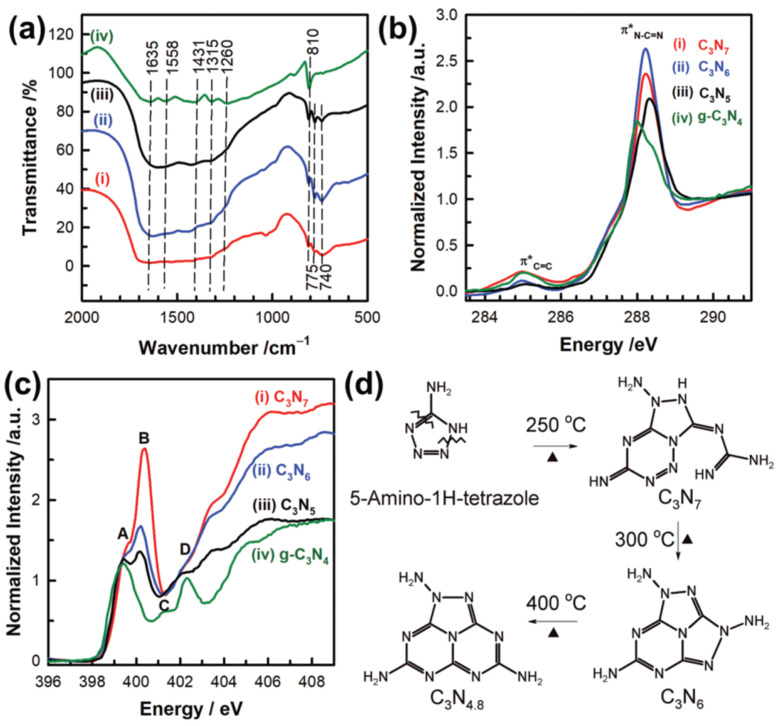
(**a**) Fourier-transform infrared spectra. (**b**) C K- and (**c**) N K-edge Near Edge X-Ray Absorption Fine Structure spectra of the (i) C_3_N_7_ and (ii) C_3_N_6_ with the reference materials of (iii) triazole-based mesoporous C_3_N_5_ and (iv) g-C_3_N_4_. (**d**) The proposed phase transition route of 5-amino-1*H*-tetrazole depending on its pyrolysis temperature [112].

The same research group, using 5-amino-1*H*-tetrazole and applying different pyrolysis temperatures, synthesized a group of materials with varying ratios of the percentage of C to N atoms, yielding C_3_N_7_, C_3_N_6_, and C_3_N_4.8_ (Figure 11d) [112]. The results in Figure 11a–c show the spectroscopic performances of the materials pyrolyzed at different temperatures. These properties, and the correlations with the electrochemical studies, made it possible to establish the effect of core structures of the carbon nitrides on their ORR activity. The ordered mesoporous C_3_N_7_ presented better ORR performances (onset potential: 0.81 V vs. RHE, electron transfer number: 3.9 at 0.5 V vs. RHE) than g-C_3_N_4_ and the ordered mesoporous C_3_N_6_. Despite the lower electronic conductivity and worse textural parameters of C_3_N_7_ compared with C_3_N_6_, C_3_N_7_ showed higher catalytic activity. These studies suggest that ORR activities can be improved by designing the structure of the carbon nitride core and controlling its chemical composition.

Li et al. proposed the application of a CNT-based composite as an efficient catalyst of the oxygen evolution reaction (OER) and ORR [116]. The high-temperature pyrolysis process applied to a mixture of glucose, urea, and CNT substrates led to the production of composites, namely N-doped mesoporous hybrid carbon nanoplates/CNTs. The resulting hybrids were used as electrode materials for the OER. They were characterized by high electrocatalytic activity with a low onset potential (1.50 V vs. RHE) and an exceptional overpotential of only 320 mV at 10 mA cm^−2^. The electrode material used for the ORR showed similar catalytic efficiency to the OER but a 20% higher value than commercially available Pt/C.

Selective oxidation of H_2_S to sulfur is an efficient process for industrial applications and environmental requirements to reduce the residual H_2_S in the Claus technology to an ultralow content (<0.1 ppm) before releasing the off-gas into the atmosphere (Table 5). Xu et al. reported that phosphate-modified N-doped mesoporous carbon/CNT composites were applicable in this process. The material presented a high sulfur selectivity of 91.3% with an excellent normalized sulfur formation rate (λ_cat_) of 503 g_sulfur_·kg_cat_^–1^·h^–1^, which was comparable with the most active carbon-based and metal–oxide catalysts ever reported [117].

Carbon/CN composites can also be used as photocatalysts (Table 5). Long et al. reported fullerene C_60_-based g-C_3_N_4_ composites prepared by calcining urea and C_60_ nanorods formed by a liquid–liquid interfacial precipitation method [119]. The obtained material exhibited efficient photocatalytic activity, i.e., better solar energy utilization, efficient transfer, and separation of photoinduced charge carriers. The optimum photocatalytic H_2_ evolution rate under visible light irradiation reached 8.73 μmol h^−1^ (with a quantum efficiency of 5.10%), about 4.7 times as high as pure g-C_3_N_4_.

### 5.3. Other Applications of Mesoporous Carbon/CN Composites

Among other applications (Table 6), capacitive deionization (CDI) is among the most useful and efficient. This technology of water purification consumes little energy to produce potable water. For improved deionization performance, the proper design of the electrode material is crucial. It should have a porous structure, good conductivity, and wettability. Graphene-based N-doped carbon spheres meet this expectation, which makes them highly efficient electrode materials for CDI applications [120]. Feng et al. reported the preparation of a 3D hierarchically porous composite containing graphene-supported N-doped carbon, which was used as an electrode material for CDI [121]. The composite was fabricated using a simple template-direct method using polystyrene spheres coated with polyacrylonitrile as the core–shell structures and as templates. Due to the hierarchically porous structure of the material, the fast transport of analyte ions was facilitated. Moreover, fast charge transfer was obtained due to the interconnected conductive networks of graphene sheets. As a result, a stable CDI performance of 25.5 mg g^−1^ salt adsorption capacity was achieved, paving the way for practical applications in water purification and environmental engineering.

Carbon composites can also find application as proton-exchange membrane fuel cells [125], single-carbon-atom-level molecular discrimination of carboxylic acid [126], or flue-gas desulfurization [38] (Table 6). Song et al. proposed synthesizing composite material as a promising high-performance adsorbent for flue gas desulfurization. The obtained material consisted of N-doped mesoporous carbons and small amount of N-doped CNTs, delivering an average SO_2_ adsorption capacity of 21.2 mg g^−1^ [38]. A multilayer cathode composite of activated carbon fibers, reduced graphene oxide, and ordered mesoporous carbon found application in removing phthalic acid esters using an electro-Fenton process [127]. When this composite was applied as an electrode, the reduction of oxygen to generate hydrogen peroxide was promoted from 31 to 85 mg L^–1^, and the current efficiency of the electro-Fenton process was also improved from 25% to 40% [127].

## 6. Conclusions

Over the last decade, numerous studies have been conducted on synthesizing carbon/CN composites with a mesoporous nanostructure. This article discusses the latest methods of composite synthesis, taking into account their physicochemical properties and comparing them in light of their applications. Carbon composites containing a polymer component and CNs are among the most promising combinations. Despite both components being organic, the versatility of these composites is genuinely inspiring. The unique morphology of both materials (polymer chains and nanostructured carbon) allows for the combination of different physicochemical properties within one material.

Further, the pyrolysis process changes the organic components, which are often non-conductive polymers, into a carbon/CN system of composites. The polymer chain gives the material a mesoporous character, adding versatility and the potential for creative applications. Consequently, mesoporous carbon/CN composites exhibit many advantages, such as improved thermal conductivity, tensile strength, electrical conductivity, wear resistance, and a better modulus of elasticity, compared with the starting substrates: polymer and CNs. Especially promising in electrochemistry and electrocatalysis are composites containing nanostructured carbon that should also be characterized by high 3D pore organization.

Additionally, the general advantages of mesoporous carbon/CN composites include low production costs, a variety of structures, and a combination of mechanical strength and lightness that conventional materials, such as metals, cannot match. In addition, the ease of obtaining the simultaneous design of various sizes and shapes of carbon materials and the ease of doping with heteroatoms makes them attractive materials for the discussed areas. However, it should be emphasized that classic carbon materials, such as activated carbon, carbon aerogels, and carbon black, meet these criteria but, on the other hand, usually have low activity, stability, and oxidation resistance. To achieve the appropriate material parameters, it is crucial to combine them appropriately in the composite by designing the proper material structure, which is considered to be an elemental composition and a homogeneous order or to create pores in 3D. The correlations among the porous structures of materials, heteroatom doping, and electrochemical or catalytic efficiency, including activity, selectivity, and stability, should also be considered.

Considering these points above, the corrugated properties of materials, their chemical composition, the arrangement of atoms with each other, their homogeneous distribution in 3D, and the types of bonds between atoms, etc., are of particular importance. In the case of porous carbon materials used in catalysis, the main limitations limiting the achievement of high catalytic (electro- and photo-) performance are a random pore structure with dominant microporosity, an uneven morphology, a high content of uncontrolled defects and impurities, low oxidative resistance to chemical reactions, and inefficient electron and heat transport. On the other hand, in the case of applications in electrochemistry, the parameters of the electrode material are critical, such as a large conductive surface area with reasonably high electrochemical cycling stability, fast charge–discharge processes, reasonably low self-discharging, high thermal stability, and well-defined porosity. Due to the above, it is therefore necessary, first of all, to ‘design’ materials for specific applications.

Achieving high process efficiency or designing devices with appropriate operating parameters and high efficiency is a highly complicated process. First, however, it is necessary to search for materials with optimized properties that can be used in specific areas. Therefore, it is essential to ‘design’ highly efficient materials with enhanced properties. While mesoporous carbon/CN composites have already found applications in catalysis and electrochemistry, the need for new solutions in the synthesis process is a challenge that should engage us. In this case, it is still necessary to search for new solutions to simplify the synthesis process, reduce production costs, enable large-scale industrial applications, or make the process more environmentally friendly.

It seems that such an effective solution is to use tools such as cheminformatics and machine learning, which enable the ‘design’ of materials with the desired properties rationally. For example, it is possible to include tools that allow us to select substrates with high potential for synthesizing the product, giving a material with the desired properties. Machine learning may be applied to predict the properties and optimize the substrates to design nanocomposites for electrochemistry and electro- and photocatalysis. Such a comprehensive approach in current materials chemistry seems to be a solution that will be used more often to optimize both the parameters of materials and the entire process of their production.

## Figures and Tables

**Table 1:** Advantages and disadvantages of templating methods.

	Hard-Template Method	Soft-Template Method
Advantages	Efficient, repeatable process Great control over the structure and geometry of pores thanks to the precise design of the template High surface areas and pore volume, tunable pore diameters, and high crystallinity of mesoporous carbon	Easy template synthesis, relatively cheap commercially available templates (e.g., pluronic F-127) Simple and environmentally friendly process, and the templates are thermally removable High specific surface area and a network of multiple-scale channels of mesoporous carbon
Disadvantages	Tedious process of template synthesis, expensive templates (e.g., SBA-15) Multiple steps, expensive, time-consuming process, harsh conditions to remove templates (e.g., HF) Possibility of pores collapsing after removal of the template	Large amounts of solvent are needed (for sufficient mixing of the reagents and template) Possibility of thermal decomposition of the material before the completion of the carbonization process

**Table 3:** Applications of mesoporous carbon/CN composites as energy storage devices.

Carbon Nanostructure	Precursor of Mesoporous Carbon	Template	Pore-Forming Agent	T (°C)	Surface Area (m^2^ g^−1^)	Pore Volume (cm^3^ g^−1^)	Pore Size (nm)	Hetero-Atom (%)	Capacity (mAh g^−1^)	Applications	Ref.
Graphene	n-Butanol, S	Hard	P123, TEOS	850	381	0.27	3.0–4.2	S	1159	Li-S batteries	[90]
Graphene	SiO_2_/MF, S	Hard	SiO_2_	800	879	2	-	N (18.6) S (70 wt%)	880.8		[91]
CNTs	Emim-dca, S	-		800	-	-	<8	N	1558.6		[87]
Graphene	Chitin, urea, g-C_3_N_4_, S	-	-	800	342	-	2–5	N S (78 wt%)	1130		[89]
Graphene	Aniline, phytic acid	-	-	800	683	0.26	-	N (4.57) P (5.07)	1469		[92]
CNTs	Macadamia nut shells, S	-	-	900	-	-	-	S	1253		[68]
Graphene	m-Aminobenzene sulfonic acid	-	-	900	185	-	3	N (4.07) S (1.28)	1355		[71]
Graphene	Resorcinol, HCHO, ammonia, S	Hard	TPOS	800					973		[88]
CNTs	Resorcinol, HCHO, ethylenediamine	Hard	TEOS	700	749	0.90	~2.7	N	conductivity 1.54 S cm^−1^	Al-Se batteries	[37]
CNTs	Dicyandiamide	-	-	800	1685	-	-	N	1840	Li-O_2_ batteries	[93]
CNOs	Nitric acid	-	-	550	406	-	20.0	N	12,181		[94]
Fullerene C_60_	Sucrose	Hard	SBA-15	900	808	1.5	3.6	-	1299	Li-ion batteries	[42]
Graphene	3-Aminopropyl triethoxysilane	Hard	TEOS	750	224	-	3.8	N	1368		[95]
CNTs	Polyvinyl alcohol, starch	-	-	700	982	0.48	2.0	-	743		[96]
CNTs	Dopamine hydrochloride	-	-	800	321	-	~3.7	N (1.6)	-	Zn–air batteries	[86]
CNTs	Resorcinol, HCHO	Soft	F-127	800	146	-	~3	-	203.6	Na-ion batteries	[23]
CNTs	Resorcinol, HCHO	Hard	TEOS	800	1045	1.09	8.3	-	1000	Na/K-ion batteries	[97]
CNTs	Aniline, (NH_4_)_2_S_2_O_8_	Hard	SiO_2_	900	786	1.66	7–22	N (7.3)	700	Flexible sulfur electrodes	[35]
CNTs	Lignin	-	-	900	1050	1.55	39	-	905	Lithium storage	[98]
Graphene	Resorcinol, HCHO	Hard	TEOS, IL	800	740	0.87	2.7	N (5.5)	347.3	Energy storage systems	[49]
Fullerene C_70_	1-Chloronaphtalene	Hard	SBA-15	900	586	0.79	10.1	-	C*s* = 172 F g^−1^		[41]
CNTs	Dopamine, TMB	Soft	F-127	800	768	0.57	6.9	N (6.9)	-		[58]
CNTs	Phenol, HCHO	Soft	F-127	600	-	-	-	-	C*s* = 20 F g^−1^		[99]
CNOs	Resorcinol, HCHO	Sof	F-127	850	700	1.14	13.7	-	-		[33]
Fullerene C_60_	1-Chloronaphtalene	Hard	SBA-15	900	680	0.85	10.6	-	C*s* = 116 F g^−1^	fuel cell	[40]

Abbreviations: TEOS—tetraethyl orthosilicate; TPOS—tetrapropyl orthosilicate; TMB—3,3′,5,5′-tetramethylbenzidine; Emim-dca—1-ethyl-3-methylimidazolium dicyanamide; IL—ionic liquid.

**Table 4:** Applications of mesoporous carbon/CN composites as electrochemical sensors.

Carbon Nanostructure	Precursor of Mesoporous Carbon	Template	Pore-Forming Agent	T (°C)	Surface Area (m^2^ g^−1^)	Pore Size (nm)	Heteroatom	Linear Range	Limit of Detection	Ref.
Graphene	Polydopamine	Hard	TEOS	800	-	-	N	0.5–400 mM for HQ) 1–300 mM (for CC) 3–200 mM (for RC)	0.15 mM 0.3 mM 1.0 mM	[101]
Graphene	Resorcinol, HCHO, boric acid, CTAB	Hard	TEOS	700	1239	6	N B	0.0915–103 μM (for X) 0.0822–128 μM (for G)	0.0503 μM 0.0462 μM	[102]
Graphene	Resorcinol, HCHO, CTAB	Hard	TEOS	800	-	-	N	0.5–189 mM (for rutin)	0.05 mM	[48]
Fullerene C_60_	Sucrose	Hard	SBA-15	900	1302	3.21	-	-	-	[103]
Graphene	Phenol, HCHO	Soft	F127	700	514	3	-	10 nM–10 μM (for doxorubicine)	1.50 nM	[57]

Abbreviations: CTAB—cetyltrimethylammonium bromide; TEOS—tetraethyl orthosilicate; HQ—hydroquinone; CC—catechol; RC—resorcinol; X—xanthine; G—guanosine.

**Table 5:** Catalytic properties of heteroatom-doped mesoporous carbon/CN composites.

Carbon Nanostructure	Precursor of Mesoporous Carbon	Template	Pore-Forming AGENT	T (°C)	Surface Area (m^2^ g^−1^)	Pore Volume (cm^3^ g^−1^)	Pore Size (nm)	Heteroatom (%)	Feature	Ref.
Application in the ORR										
Graphene	Dopamine, mercapto-ethanol	-	-	800	273	0.33	3.6	N (4.1) S (6.1)	Double-layer capacitance 11.1 mF cm^−2^	[110]
Graphene	2-Fluoroaniline	Hard	FeOOH	800	821	0.66	-	-	Current density 6.1 mA cm^−2^	[111]
Graphene	5-Amino-1*H*-tetrazole	Hard	P123 TEOS	540	301	-	3.4	N	Current density 11.1 mA cm^−2^	[109]
Graphene	5-Amino-1*H*-tetrazole	Hard	P123 TEOS	250 300	114 167	-	4.4 3.3	N	Current density 8.2 mA cm^−2^ 7.5 mA cm^−2^	[112]
Graphene	*m-*Phenylene-diamine	Soft	PS-b-PEO	900	812	0.69	19	N (2.2)	Current density 5.2 mA cm^−2^	[113]
Graphene	*m*-Phenylene-diamine	Soft	P123 or F127	800	420	-	8	N (2.6)	Current density 4.8 mA cm^−2^	[114]
Graphene	*m*-Amino-thiophenol	Soft	PS-*b*-PEO	900	799	0.94	9.4	N (3.4) S (0.4)	Current density 5.66 mA cm^–2^	[115]
CNTs	Melamine, 2-thenaldehyde	-	-	900	407	-	5.44	N (1.9) S (0.23)	-	[67]
CNTs	Urea, glucose	-	-	800	594.1	0.58	2–50	N (8.5)	ORR and OER activity with a low onset potential	[116]
Application in selective H_2_S oxidation										
CNTs	D-glucose, citric acid, (NH_4_)_2_CO_3_	-	-	900	537	0.73	6.1	N (4.0–9.1)	-	[32]
CNTs	D-glucose, citric acid, (NH_4_)_2_CO_3_	-	-	800	330	-	-	N (4.0–5.4) P (0.5–3.3)	-	[117]
Application in catalysis and photocatalysis										
CNTs	*D*-glucose,citric acid, (NH_4_)_2_CO_3_	-	-	900	516	-	~4	N (19.5)	-	[118]
Fullerene C_60_	Urea	-	-	600	117.47	-	2.0–6.0	N	-	[119]

**Table 6:** Other applications of mesoporous carbon/CN composites.

Carbon Nanostructure	Precursor of Mesoporous Carbon	Template	Pore-Forming Agent	T (°C)	Surface Area (m^2^ g^−1^)	Pore Volume (cm^3^ g^−1^)	Pore Size (nm)	Heteroatom (%)	Feature	Ref.
Application in capacitive deionization										
Graphene	Dopamine	Hard	TEOS	800	1270	1.6	8–10	N (4.2)	C*s* = 125.7 F g^−1^	[4]
Graphene	Bean protein	-	-	850	1286	1.15	3.58	N (1.93)	C*s* = 370 F g^−1^ adsorption capacity 38.5 mg g^−1^	[122]
Graphene	Resorcinol, HCHO	Hard	TPOS	700	338	0.62	-	N (3.11)	C*s* = 226.5 F g^−1^ electrosorption capacity 17.8 mg g^−1^ in 500 mg L^−1^ NaCl	[120]
CNTs	Poly(vinylidene fluoride)	-	-	800	905	0.48	2–10	-	Electrosorption capacity 15.1 mg g^−1^ in 500 mg L^−1^ NaCl	[123]
Graphene	Polyacrylonitrile, polystyrene	Soft	PS spheres	700	650	0.16	10.3	N	Electrosorption capacity 25.5 mg g^−1^ in 500 mg L^−1^ NaCl	[121]
Application in proton-exchange membrane fuel cells										
Fullerene C_60_	1,2,4-Trinitrobenzen	Hard	KIT-6 SBA15	900	310.5 100.0	-	2.5 3.5	-	-	[124,125]
Application in single-carbon-atom-level molecular discrimination										
Fullerene C_60_	Ethylenediamine	-	-	700	655.2	0.659	3.66	N (1.2)	-	[126]
Fullerene C_70_	Ethylenediamine	-	-	700	114.1	0.257	3.88	N (1.7)	-	[126]
Application in flue gas desulfurization										
CNTs	Melamine, phenolic resin	Hard	MgO	700	223	-	20	N (6.1)	SO_2_ capacity 21.2 mg g^−1^	[38]
Application in the electro-Fenton process										
Graphene	Active carbon fiber, resol	Soft	F127	800	533	0.45	3.8	-	Electroactive surface area 486 cm^2^ g^–1^ Electron transfer resistance 8.60 Ω	[127]
Electrical applications										
CNTs	Resorcinol, HCHO	Soft	NaDBS	1050	1507.5	0.99	3.26	-	-	[31]

Abbreviations: PS—polystyrene; NaDBS—sodium dodecylbenzene sulfonate.
